# Breed Group Effects on Complaints about Canine Welfare Made to the Royal Society for the Prevention of Cruelty to Animals (RSPCA) Queensland, Australia

**DOI:** 10.3390/ani9070390

**Published:** 2019-06-26

**Authors:** Hao Yu Shih, Mandy B. A. Paterson, Clive J. C. Phillips

**Affiliations:** 1 Centre for Animal Welfare and Ethics, University of Queensland, White House Building (8134), Gatton Campus, Gatton, QLD 4343, Australia; 2 Royal Society for the Prevention of Cruelty to Animals Queensland, Brisbane, QLD 4076, Australia

**Keywords:** canine welfare, breed, canine cruelty, neglect, RSPCA

## Abstract

**Simple Summary:**

This retrospective study involves 107,597 dog welfare complaints received by the Royal Society for the Prevention of Cruelty to Animals (RSPCA) Queensland from 2008 to 2018. Results show that, compared to pure breed dogs, cross-breed dogs were more likely to be reported in welfare complaints. Poisoning, lack of veterinary support, abuse, and being left unattended in a hot vehicle were common complaints in pure breed dogs; while insufficient shelter, exercise and food/water, as well as overcrowding and abandonment, were more commonly reported in cross breed dogs. Utility breeds, terriers and working dogs were most likely to be reported, while toy, non-sporting breeds and gundogs were least likely to be reported. Common complaint types for utility dogs were: insufficient food/water, shelter and exercise, and poor living conditions; for terriers: abandonment, intentional abuses and killing or injuring another animal; for working dogs: insufficient food/water, shelter and exercise; for toy dogs: lack of veterinary care, overcrowding and staying in a hot vehicle alone; for non-sporting dogs: lack of veterinary care, being left in a hot vehicle unattended and poor body conditions; and for hounds: killing or injuring another animal, intentional abuses and poor body conditions.

**Abstract:**

Cruelty- and neglect-related canine welfare concerns are important welfare and social issues. Dog breed has been identified as a risk factor for bad welfare, and yet its role in different types of canine welfare concerns has not been fully investigated. We conducted a retrospective study of 107,597 dog welfare complaints received by RSPCA Queensland from July 2008 to June 2018. The breed of the dog involved in the incident was either recorded as stated by the complainant or by the inspector attending the case. Dog breed was divided into groups following the Australian National Kennel Club nomenclature. Dogs of a non-recognised breed were more likely to be reported in welfare complaints than recognised breed dogs. Recognised breed dogs had a greater risk of being reported with poisoning, lack of veterinary support, abuse and being left unattended in a hot vehicle; while non-recognised breed dogs had greater risk of being reported with insufficient shelter, exercise and food/water, as well as overcrowding and abandonment. Utility breeds, terriers and working dogs were most likely to be reported, while toy, non-sporting breeds and gundogs were least likely to be reported. Common complaint types for utility dogs were: insufficient food/water, shelter and exercise, and poor living conditions; for terriers: abandonment, intentional abuses and killing or injuring another animal; for working dogs: insufficient food/water, shelter and exercise; for toy dogs: lack of veterinary care, overcrowding and staying in a hot vehicle alone; for non-sporting dogs: lack of veterinary care, being left in a hot vehicle unattended and poor body conditions; and for hounds: killing or injuring another animal, intentional abuses and poor body conditions. Breed groups rather than breeds may be the best method of breed identification in a public reporting system as they group similar breeds together, and as our research shows, they relate to types of animal welfare complaints. Understanding the relationship between breed group and canine welfare complaints may help authorities improve public education programs and inform decision-making around which breed a new owner should choose.

## 1. Introduction

Animal cruelty can be defined as any socially [1] or legally [2] unacceptable behaviour causing unnecessary pain, discomfort or distress to animals. It is an important social issue, affecting not only animals but also the entire society, for example, there appears to be a strong relationship between animal cruelty and other criminal activities, such as domestic violence [3]. Animal cruelty can happen to any animal, and the dog (*Canis familiaris*) is one of the most common species reported for suspected animal cruelty [4]. 

For over 15,000 years, dogs have been domesticated to fit into our society with humans intentionally taming them, which facilitates the development of close bonds between humans and dogs [5,6]. According to the Australian National Kennel Club (ANKC), there are 208 recognised breeds that are categorised into seven breed groups—toys, terriers, gundogs, hounds, working dogs, utility, and non-sporting—on the basis of their physical characteristics, temperaments, behaviours, and functions [7]. For example, greyhounds were originally bred to chase and hunt in the Egyptian deserts 5000 years ago, and are categorised as hounds by the ANKC [7,8]. Border collies were selected for their sheep herding abilities in western Europe, northern England and Scotland over 100 years ago, and are classified as working dogs by the ANKC [7,9,10]. However, the original selection traits are often of little value today and their purpose has broadened to become purely aesthetic or related to entertainment (including gambling), causing them to experience some breed-specific mistreatments. For instance, greyhounds [11] and huskies [12] are used in the racing industry, and greyhounds have been widely reported to experience welfare issues associated with their training, living conditions and injuries during racing. The greyhound racing industry has been associated with “live baiting”, where a live animal, such as a chicken or possum, is used as a lure to train greyhounds to race [11,12,13]. Border collies are popular for companionship, but their herding instincts may not be fulfilled in a home environment, which predisposes them to behavioural problems, such as bike or runner chasing, and can finally lead to unsuccessful ownerships [14]. Pit bull type dogs, such as the American pit bull terrier, American Staffordshire terrier and Staffordshire bull terrier are often thought of as aggressive [15]. These dogs may be made to participate in illegal dog fights or used for pig hunting, and animal management laws may be biased against them. In Australia, the importation of American pit bull terriers is banned [16] and the law requires all American pit bull terriers currently in Australia to be sterilised. In addition, these dogs must be kept strictly confined when at home, and when taken out be muzzled and wear easily identifiable collars [15,17]. Similarly, in America, some states (e.g., Indiana and Louisiana) require pit bull owners to obtain a special license and maintain $100,000 to $300,000 in liability insurance to cover any potential injuries caused by the dogs [18]. Many studies highlight the negative welfare that may be experienced by racing and fighting dogs [17,19]. The welfare issues they experience are not only breed specific, but are directly related to specific industries (e.g., dog racing and fighting) [20]. However, neglect-related issues, such as failing to provide suitable food and water, veterinary support and suitable living conditions, are more common, yet less discussed [3,21]. To our knowledge, there has been little consideration of the correlation between canine breed and different forms of animal welfare concerns, particularly those related to neglect. 

Accurate breed identification is useful in many areas including in shelters, veterinary clinics, research, and even the media [22]. How dogs are identified influences the way they are perceived and how people interpret their behaviours [23,24,25]. For instance, dogs identified as terriers, especially American pit bull terriers and Staffordshire bull terriers are perceived as playful, curious, fearless, chase prone and aggressive; however, dogs identified as toy breeds are seen as sociable [25]. Current breed identification in the majority of facilities is based on observation, but such visual identification of breed is problematic and often inaccurate [22,26]. In a laboratory-based experiment, 986 people engaged in dog-related professions were asked to visually identify the breed of 20 dogs using video clips. The visual identification was later compared with DNA identification. The results showed that over 50% of participants failed to visually identify dog breeds that matched the DNA identification, and agreement by over 50% of participants was only found with 35% (*n* = 7/20) of dogs [22]. Another study exploring inter-observer agreement among shelter staff differentiating pit-bull-type dogs versus non-pit-bull-type dog revealed moderate reliability (76%–83%) [26]. Consequently, a better breed identification method is needed, and identifying dogs per group or type (e.g., pit-bull type), rather than by specific breed, may result in higher agreement among individuals and be more useful. 

In Queensland, Australia, animals are protected by the Animal Care and Protection Act 2001 (ACPA) [2]. This state-based legislation appoints inspectors, some of whom are employed by the Royal Society for the Prevention of Cruelty to Animals, Queensland (RSPCA Qld), to investigate potential breaches of, and enforce compliance with, the Act [2]. There are two main offences under the ACPA: failure to fulfil duty of care responsibilities and cruelty. There are a number of other specified offences. The Act recognises that a person who has charge of an animal owes that animal a duty of care. Failure to provide such care is the basis of the “breach of duty of care” offence. This offence covers such actions as not providing sufficient food, water, exercise, veterinary care and suitable living conditions. It is not only the owner that has a duty of care towards an animal. Anyone who is even temporarily in charge of an animal has a duty of care. The second major offence is “animal cruelty” and according to Section 18 of the Act, cruelty describes any action that causes unjustifiable and unnecessary physical and mental discomfort to animals, inappropriate confinement or transport, unreasonable injuries and inhumane death [2]. A cruel act can be committed by anyone towards an animal, whether it is their own animal, another domestic animal or even a wild animal [2]. It is important to note, that under the ACPA, the intention of a person to be cruel is not a necessary element of a cruelty offence to be proven in Queensland. If an action carried out by a person causes pain and suffering and the action was intentional, the person may be charged with cruelty. The intention to carry out the action must be proved but not the intention to be cruel. If a lack of action deprives an animal of its fundamental needs, then the person who has a duty of care towards the animal may be charged with a breach of their duty of care or cruelty depending on the circumstances. Intention may be considered during sentencing however [2]. Other offences under the Act include unreasonable abandonment or release, the carrying out of prohibited surgical procedures (e.g., tail docking, ear cropping, debarking, etc.), being involved in, or having items used for, a prohibited event, such as dog or cock fighting, and allowing an animal to injure or kill another animal [2]. 

The public can report suspected welfare concerns to the RSPCA via a “Cruelty Complaints” telephone number, which operates 24 h a day, seven days a week, or by email. In addition, complaints can be made by veterinarians and veterinary nurses, council officers, and other government and non-government employees visiting a location as part of their duties. Finally, animals entering a shelter may trigger an investigation if cruelty or neglect is suspected.

In this study, we aimed to evaluate whether breed was an important factor in relation to canine welfare concerns. This report is the second in a series relating to the analysis of RSPCA Qld canine welfare complaint data [21,27]. We hypothesized that certain breeds would have a higher risk of being reported. We also hypothesized that some breeds would be at higher risk of suffering specific welfare issues than others. Other risk factors, age of the dog [21] and socioeconomic status of the complainant are the subject of other papers [21,27].

## 2. Materials and Methods 

From July 2008 to June 2018, RSPCA Qld received 129,036 canine welfare complaints. Some involving more than one dog were recorded as multiple complaints sharing the same case number, while others were recorded as one complaint with multiple animals. To avoid sample bias due to multiple entries, we only retained the first complaint of case numbers with multiple entries, discarding 21,439 entries as a result. There remained 107,597 canine welfare complaints for this retrospective study. The data analysis was originally undertaken on the entire dataset and then redone with the reduced number. Finding the complaint distribution and demographics to be similar, we opted for the reduced dataset to avoid problems with pseudoreplication. Animal welfare complaints that fell within the geographical zone of responsibility of RSPCA Qld (determined by a Memorandum of Understanding between RSPCA Qld and Biosecurity Queensland, the Government Department tasked with the administration of ACPA) were investigated by RSPCA Qld inspectors. All other complaints were referred to Biosecurity Queensland to be investigated by their inspectors. However, all complaints coming into RSPCA Qld were included in this analysis.

All complaints were recorded in ShelterBuddy^®^ (RSPCA, Queensland, Australia), the RSPCA Qld database. The following information was requested from the reporter of each incident at the time of taking the complaint: the number of dogs involved and their age, breed(s) (if known), the “complaint code(s)”, the suburb, postcode, and in addition, the date was recorded. All cases were investigated either by RSPCA Qld inspectors (*n* = 100,432) or Biosecurity Qld inspectors (*n* = 7165). It is recognized that some of the calls did not relate to a breach of the ACPA or to a genuine welfare concern. The outcome data for these complaints was not analysed in this research. This research is focused on the complaint calls coming in to RSPCA Qld. 

Dogs were classified according to two broad age ranges, being dog and puppy, based on reporters’ interpretation. It is important to recognise that the information recorded from the complainant may be inaccurate or inaccurately interpreted, e.g., a small dog is commonly referred to as a puppy in Queensland. Records regarding breed and the number of dogs involved were based on either complainants’ initial reports or comments from trained inspectors, again recognising inaccuracies with identification of the breed. The “complaint code” was selected by the staff member receiving the call or email from a drop-down menu of 18 possible complaints (Appendix A Table A1) [21]. Multiple “complaint codes” were able to be selected for each case according to the description of what was alleged to have happened to the dog(s), and each was treated as a separate code for analysis. 

### 2.1. Dog Breeds

The distribution of breeds was compared to the breeds of registered dogs obtained from the councils of two cities situated close to the RSPCA Qld headquarters, namely Ipswich City Council and Gold Coast City Council for the same period. Any breed in our data that was documented in any of the following kennel clubs—Australian National Kennel Council (ANKC) [7], New Zealand Kennel Club (NZKC) [28], American Kennel Club (AMKC) [29] and United Kennel Club (UKC) [30]—was considered a recognised breed (RB) and was added to our breed list (Appendix A Table A2). Any breed in our data that was not recognised by at least one of the major kennel clubs listed above was classified as a non-recognised breed (N-RB), including all crossbred dogs without any identified breed. In our dataset, it was decided that if more than one dominant breed was listed, the first breed mentioned would be used. For instance, Great Dane × Bull Arab was categorized as Great Dane (Appendix A Table A2). 

To achieve a secondary representation of breed recognition, RB breeds were amalgamated into the following seven breed groups based on the breed inclusion categories of the ANKC: toys, terriers, gundogs, hounds, working dogs, utility, and non-sporting. Breeds not listed by the ANKC, but recognised by the NZKC, AMKC, or UKC, were categorized into one of the seven groups based on the description of each kennel club. Some breeds (e.g., Australian Koolie and Bull Arab), though listed by the council registrations and thus on the breed list (Appendix A Table A2), were not recognized as breeds by any major kennel club worldwide. Therefore, these breeds were categorized as N-RB. If the breed description was left blank, the dogs’ breed was considered unknown (*n* = 15,576/107,597), and these complaints were excluded from any data analysis related to breed factors. 

### 2.2. Statistical Analysis

Data was analysed using the statistical package Minitab^®^ 17.3.1. (Minitab, LLC., State College, PA, USA) Descriptive analysis was first used to investigate the distribution of RB/N-RB and the seven breed groups. Complaints reported in July 2017 and June 2018 that contained breed information provided by RSPCA inspectors (*n* = 95) were used to examine the agreement of breed identification between the complainant and inspectors. Apart from simple percent agreement measurements, Cohen’s kappa coefficient was calculated. Cohen’s kappa is a statistical method measuring agreement with qualitative assessments among different raters. It is more robust than a percentage because it considers the possibility of the agreement occurring by chance [26,31]. To examine whether RB, N-RB or certain breed groups were more likely to be reported, the study group was compared with the registration data from Gold Coast and Ipswich City councils where all owned dogs, including working dogs on farms, are required to be registered [32,33]; this was done using Pearson chi-square tests. Eighteen stepwise forward binary logistic regression models were constructed to understand how breed factors correlated with each complaint code. The binary logistic regression model is a nonlinear model using a logistic function to describe the relationship of independent variables and a dependent variable with two possible values, such as yes/no, 0/1, or healthy/sick [34]. The stepwise forward selection refers to a step-by-step method of adding the most significant dependent variable into the model [35]. To determine the effect of breed (RB/N-RB or breed group) on complaint codes, breed (RB/N-RB or breed group) was entered into the binary logistic regression model as a fixed factor, using logit models with the alpha value to enter being 0.15. Complaint codes were entered into the model as outcomes. Separate models were constructed for each complaint code with the same input variable.

## 3. Results

### 3.1. Dog Characteristics

Common breeds reported by the complainants were Staffordshire bull terrier (10.5%, *n* = 10/95), American Staffordshire terrier (10.5%, *n* = 10/95), Maltese (6.3%, *n* = 6/95), and Bullmastiff (5.3%, *n* = 5/95). Overall, the agreement between complainants and inspectors of breed identification was 23.2% (Cohen’s Kappa = 0.074, indicating a slight agreement [31]), and the agreement of breed group identification was 77.8% (Cohen’s Kappa = 0.69, indicating a substantial agreement [31]). Therefore, breed groups were used for further analyses.

In the study group, 32.7% (*n* = 35,178) of dogs were N-RB, while only 1.7% (*n* = 1733) of dogs in the Gold Coast and Ipswich councils’ data were listed as N-RB. Around 53% (*n* = 56,843) of dogs in our data and 98.3% (*n* = 99,266) of dogs in the councils’ data were of RB (Table 1). The remaining dogs (14.5%, *n* = 15,576) in our database were unspecified. Thus, there was an over-representation of N-RB and an under-representation of RB in our dataset. The most common breed group to be reported for canine welfare concerns in our dataset were terriers (28.2%, *n* = 16,030), followed by working dogs (24.8%, *n* = 14,085), utility dogs (15.6%, *n* = 8,857), toy dogs (9.2%, *n* = 5223), non-sporting dogs (8.9%, *n* = 5071), gundogs (7.8%, n=4417), and hounds (5.6%, *n* = 3160) (Table 2). The most common breed group registered by the city councils were also terriers (22.2%, *n* = 22,056), but followed by toy dogs (21.0%, *n* = 20,796), working dogs (17.8%, *n* = 17,637), non-sporting dogs (14.0%, *n* = 13,915), gundogs (11.6%, *n* = 11,504), utility dogs (7.8%, *n* = 7770), and hounds (5.6%, *n* = 5581) (Table 2).

### 3.2. Predispositions of RB/N-RB to Welfare Complaints

Table 1 summarizes the numbers and percentages of RB and N-RB in our data and the councils’ data, along with the overrepresentation coefficients. This coefficient is a simple method to compare the two percentages (see explanation below Table 1). Our results indicate that N-RB were at a greater risk of being reported than RB (*p* < 0.001). 

We further explored the association between RB/N-RB and different complaint codes. A logistic regression model was generated (Table 3). In the model, there were significant correlations between RB/N-RB and nine (*n* = 9/18) complaint codes. RB had significantly greater risk of being reported with the following complaint codes, listed in increasing order of odds ratio (OR): baiting/poisoning (OR = 0.36, *p* < 0.001), no treatment (OR = 0.59, *p* < 0.001), cruelty (OR = 0.95, *p* = 0.004), and hot animal in car (OR = 0.95, *p* = 0.043). Meanwhile, N-RB had significantly greater risks of experiencing the following complaint codes, listed in declining order of odds ratio (OR): no exercise/confined/tethered (OR = 1.32, *p* < 0.001), overcrowding (OR = 1.32, *p* < 0.001), abandonment (OR = 1.25, *p* < 0.001), no shelter (OR = 1.19, *p* < 0.001), and insufficient food and/or water (OR = 1.08, *p* < 0.001).

### 3.3. Predispositions of Breed Groups to Welfare Complaints

When we compared the numbers of different breed groups in our data with the councils’ data (Table 2), the following breed groups were over-represented in our data, listed in declining order of overrepresentation coefficient (*p* < 0.001): utility, working dogs and terriers. The following breeds in our database listed in increasing order of overrepresentation coefficient were underrepresented (*p* < 0.001): toys, non-sporting and gundogs. 

In the regression model (Table 3), twelve (*n* = 12/18) complaint codes were predicted by breed group. Detailed results related to breed groups are summarized in Table 4. Toy dogs were more likely to be the subject of: no treatment, hot animal in car and overcrowding; terriers to: abandonment, cruelty, and knowingly allow an animal to kill/injure another; utility dogs to: insufficient food and/or water, no shelter, no exercise/confined/tethered and poor living condition; non-sporting dogs to: no treatment, hot animal in car and poor dog condition; hounds to: knowingly allow an animal to kill/injure another, cruelty and poor dog condition; and working dogs to: no exercise/confined/tethered, no shelter and insufficient food and/or water.

## 4. Discussion

### 4.1. Breed Identification

The agreement of breed recognition between the general public and RSPCA inspectors was very low, which is in line with a previous study demonstrating inconsistent breed identification [22]. In light of the inconsistency, instead of assigning a breed for each dog, a potential alternative is to group them by function or more general characteristics into a recognised breed group [36]. Various kennel clubs have adopted similar concepts in their breed group criteria. For instance, in the ANKC, dogs that are originally bred to work with livestock are classified as working dogs, and those developed to assist hunters in retrieving game are classified as gundogs [7]. In this study, breed information provided by the general public and RSPCA inspectors was used to categorise dogs into ANKC breed groups. Consequently, the agreement increased from 23.2% to 77.8%, with substantial reliability (Cohen’s Kappa = 0.69) between the public and trained inspectors. This result suggests that both the general public and inspectors are able to recognize some important and obvious characteristics of the dogs. Hence breed groups, rather than breeds, may be a better and more practical way of classifying breed for shelter research.

### 4.2. RB versus N-RB

This study examined the relationship between breed and canine welfare complaints. Specifically, we examined RB/N-RB and different breed groups with respect to their predisposition to be reported for welfare problems. We found there was a greater proportion of RB in the councils’ data compared with RSPCA’s, which might reflect a low rate of registration of N-RB, mainly crossbred dogs, in the two Queensland regions or that RB dogs were less likely to be involved in poor welfare or cruelty. These findings are supported by a previous study showing that crossbred dogs are at a higher risk of non-accidental injuries as a result of physical abuses, such as beating, throwing and burning, than pure bred dogs [37]. A further analysis of different complaint codes revealed that RB were predisposed to complaints related to “gaming”, where owners allowed these dogs to engage in racing, fighting and blood sports. The number of dog fight cases was relatively small [21], so we should interpret them cautiously, although it is likely that dog fighting occurs more frequently than is reported. Credible and actionable evidence of such events is rarely received. One surprising finding was RB dogs were reported more often than N-RB for not receiving adequate veterinary care. Previous research into pet ownership and attitudes to pet care found that owners of shelter-acquired pets, usually mixed breed [38], took their animals for veterinary care more often and were equally willing to spend over $1000 on medical treatment for their pets [39] than pets acquired by other means. Finally, our data suggest that many N-RB dogs are not registered, which may make them more likely to be surrendered to a shelter or abandoned when medical care is required. Previous studies have reported higher rates of surrender of N-RB dogs [38]. N-RB dogs were more likely to be involved in complaints related to husbandry practices and abandonment. 

### 4.3. Breed Groups

RB dogs were divided into breed groups and strong correlations between the groups, characteristics of the breeds and reasons for being reported were observed. For instance, toy dogs are small and possibly travel with owners more often, and thus, as found in our study, were more likely to be left alone in a car in hot weather. Previous research reports that smaller breeds of dogs are popular in Australia [40], which might help explain the number of complaints about toy dogs being left unattended in hot vehicles, as reported previously [21]. However, increased awareness of the dangers for dogs in hot cars through regular campaigning on this issue may also explain the high number of reports received [41]. Many terriers, especially those with some pit bull type characteristics (e.g., Staffordshire bull terrier, American Staffordshire terrier, and pit bull terrier), are considered aggressive and dangerous [15], therefore would be predisposed to being reported for abandonment, dog fights and cruelty [17,19,37,42]. Finally, utility and working dogs are mostly bred for guarding, rescuing or herding functions [7], and are generally energetic and require exercise [43,44]. These breeds were reported for not receiving adequate exercise. In contrast, toy breeds were the least likely to be reported for insufficient exercise, which is in agreement with previous research that found that smaller dogs were likely to have their exercise needs met even though they were walked less frequently [45].

### 4.4. Practical Application

This study provides fundamental information about the relationship between breed groups and various types of welfare complaints in dogs. The information can be used to develop education campaigns to increase awareness of what is involved in adequately and appropriately caring for dogs. Specific breeds have specific needs with respect to, for example, exercise requirements and cognitive enrichment to ensure their welfare is good. Information could be made available to prospective new owners of specific breeds to improve their understanding of the breed and its care requirements. Such information could also inform decision-making around breed choice as requirements of the breed could be matched with the ability of the owner to provide these needs.

### 4.5. Limitations and Need for Future Research

This was the first study providing fundamental information of the relationship between dog breed and welfare complaints made to a welfare organization with the responsibility of administering the Animal Welfare Act. Future research could focus on common breeds and explore the welfare issues in more detail. 

There were several limitations of this present study. First, N-RB dogs contributed 32.7% of our dataset but only 1.7% of the total population in the councils’ data. This major difference might indicate that N-RB dogs were indeed more susceptible to animal welfare concerns, but might also be affected by: (1) the difficulty of breed recognition [22], (2) the potentially different criteria of breed classification in our data and the councils’ data and (3) the possibly lower registration rate of N-RB. Second, complaint codes were made based on public reports, which are likely to be inaccurate, at least in some cases. Third, we compared our data with reference data from the Gold Coast and Ipswich City Councils, urban areas in South East Queensland. The RSPCA cases were collected from a broad geographical area including both urban and rural areas, which may cause some regional bias. There were only 0.76% (*n* = 814/107,597) of cases from the Gold Coast and Ipswich regions. If we only compare the cases in these two regions, our results may be skewed by some less-common breeds that are either reported in our data or the councils’ registration. Our data cover the Queensland regions along the East Australian seashore, which are nearly identical to the previous research [46]. Therefore, we decided to use similar methods by comparing our entire data with the councils’ data. Given these limitations, the data reported here included canine welfare complaints only in Queensland, and national or global generalization should be made with caution.

## 5. Conclusions

Dog identification classified on the basis of breed groups rather than specific breed had a higher agreement between the public and shelter staff, and thus may serve as a better method of describing dogs involved in welfare reports. N-RB dogs, mainly crossbred dogs, were significantly more likely to be reported for alleged animal welfare concerns, especially poor living conditions and abandonment than RB. In addition, the characteristics of specific breeds, such as size, physical traits and exercise demands, were correlated to the reported complaints. Our results can help to improve public education and awareness raising. Finally, future studies are encouraged to explore in more detail the relationships between breed and welfare issues in dogs.

## Figures and Tables

**Table 1:** Distribution of RB and N-RB in our study group, and in the Ipswich City Council and Gold Coast City Council registrations, with the overrepresentation coefficient (both RB and N-RB were significantly different (chi-square *p* < 0.001).

Breed	Study Group	Ipswich and Gold Coast	Overrepresentation Coefficient ^a^
N-RB	35,178 (32.7%)	1733 (1.7%)	19.24
RB	56,843 (52.8%)	99,266 (98.3%)	0.54
Unknown	15,576 (14.5%)	0 (0%)	-- ^b^
Total	107,597 (100%)	100,999 (100%)	

^a^ Percent of breeds in our study/percent of breeds in the councils’ registrations. 1.00 signifies equal representation in our database, and Ipswich and Gold Coast City Councils registrations. ^b^ Unable to calculate the overrepresentation coefficient because there was no dog with an unknown breed in the councils’ data.

**Table 2:** Distribution of each breed group in our study, and in Ipswich City Council and Gold Coast City Council registrations, with the overrepresentation coefficient.

Breed Groups	RSPCA	Ipswich and Gold Coast	Overrepresentation Coefficient ^a^	*p*-Value (Chi-Square Tests)
Terriers	16,030 (28.2%)	22,056 (22.2%)	1.27	<0.001
Working Dogs	14,085 (24.8%)	17,637 (17.8%)	1.39	<0.001
Utility	8857 (15.6%)	7770 (7.8%)	1.99	<0.001
Toys	5223 (9.2%)	20,796 (21.0%)	0.44	<0.001
Non-sporting	5071 (8.9%)	13,915 (14.0%)	0.64	<0.001
Gundogs	4417 (7.8%)	11,504 (11.6%)	0.67	<0.001
Hounds	3160 (5.6%)	5581 (5.6%)	0.99	0.602
Total	56,843 (100%)	99,266 (100%)		

^a^ Percent of breeds in our study/percent of breeds in the councils’ registrations. 1.00 signifies equal representation in our database, and Ipswich and Gold Coast City Councils registrations.

**Table 3:** Odds ratio of each variable in the logistic regression model of complaint codes. The outputs of these models were different complaint codes. The input variables were N-RB/RB and breed groups.

Complaint Code	N-RB/RB *p*-Value/or (CI) ^a^	Breed Group *p*-Value
Emergency relief	-- ^c^	-- ^c^
Hot animal in car	0.043/0.95 (0.90, 1.00)	<0.001
Keeping or using animal for blooding/coursing a dog	-- ^c^	-- ^c^
Prohibition order breached	-- ^c^	-- ^c^
Causing captive animal to be injured/killed by dog	-- ^c^	-- ^c^
Poor dog condition	-- ^c^	<0.001
Overcrowding	<0.001/1.32 (1.14, 1.53)	<0.001
No exercise/confined/tethered	<0.001/1.32 (1.28, 1.36)	<0.001
Insufficient food and/or water	<0.001/1.08 (1.05, 1.12)	<0.001
Baiting/poisoning	<0.001/0.36 (0.30, 0.43)	<0.001
Tail docking or other surgical procedure	-- ^c^	-- ^c^
No treatment	<0.001/0.59 (0.57, 0.62)	<0.001
Dog fighting or other prohibited offence	0.134/0.79 (0.58, 1.08)	-- ^c^
No shelter	<0.001/1.19 (1.14, 1.24)	<0.001
Poor living condition	-- ^c^	0.046
Cruelty ^d^	0.004/0.95 (0.91, 0.98)	<0.001
Abandonment	<0.001/1.25 (1.21, 1.29)	<0.001
Knowingly allowing an animal to kill/injure another	0.111/0.86 (0.71, 1.04)	<0.001

^a^ Odds ratio refers to N-RB relative to RB; ^b^ Odds ratio and the 95% confidence interval (CI) of each breed group for every complaint code are presented in Table 4; ^c^ Breed factor (N-RB/RB or breed group) was not selected in the logistic regression model; ^d^ A person was reported to have abused an animal.

**Table 4:** Odds ratio and the 95% confidence interval (CI) of each breed group for every complaint code.

	No Treatment	Abandoned	Cruelty ^a^	Insufficient Food and/or Water	No Shelter	No Exercise/Confined/Tethered	Hot Animal in Car	Baiting and Poisoning	Poor Dog Condition	Overcrowding	Knowingly Allowing an Animal to Kill/Injure Another	Poor Living Condition
Hounds/Gundogs	1.00 (0.89, 1.12)	0.92 (0.82, 1.04)	**1.17 (1.03, 1.33)**	0.96 (0.87, 1.07)	**0.74 (0.64, 0.85)**	1.10 (0.99, 1.23)	**0.82 (0.68, 0.99)**	0.77 (0.54, 1.12)	**1.47 (1.32, 1.62)**	1.56 (0.94, 2.57)	**2.44 (1.38, 4.29)**	1.10 (0.97, 1.24)
Non-Sporting/Gundogs	**1.31 (1.19, 1.45)**	0.94 (0.85, 1.05)	**0.83 (0.74, 0.94)**	**0.88 (0.80, 0.96)**	**0.67 (0.59, 0.76)**	**0.75 (0.68, 0.83)**	**1.62 (1.41, 1.87)**	**0.67 (0.48, 0.94)**	**1.65 (1.51, 1.80)**	1.12 (0.69, 1.80)	0.55 (0.27, 1.13)	1.05 (0.94, 1.17)
Terrier/Gundogs	**1.31 (1.19, 1.45)**	1.08 (0.99, 1.18)	**1.28 (1.16, 1.41)**	1.01 (0.94, 1.09)	**0.88 (0.80, 0.97)**	1.06 (0.98, 1.15)	**1.22 (1.08, 1.38)**	**0.49 (0.37, 0.64)**	1.01 (0.94, 1.09)	0.70 (0.46, 1.06)	**1.76 (1.08, 2.85)**	1.02 (0.94, 1.12)
Toys/Gundogs	**1.50 (1.36, 1.66)**	0.92 (0.83, 1.02)	0.97 (0.86, 1.08)	**0.63 (0.58, 0.70)**	**0.41 (0.36, 0.48)**	**0.51 (0.46, 0.57)**	**2.67 (2.34, 3.05)**	0.85 (0.62, 1.16)	1.07 (0.97, 1.17)	**2.08 (1.35, 3.20)**	0.71 (0.36, 1.38)	0.98 (0.88, 1.09)
Utility/Gundogs	0.98 (0.89, 1.07)	1.03 (0.94, 1.13)	1.01 (0.91, 1.12)	**1.12 (1.03, 1.22)**	**1.13 (1.02, 1.26)**	**1.38 (1.26, 1.50)**	**0.49 (0.42, 0.57)**	**0.50 (0.36, 0.68)**	**1.45 (1.34, 1.57)**	0.76 (0.48, 1.20)	1.21 (0.71, 2.06)	**1.10 (1.00, 1.21)**
Working Dogs/Gundogs	0.93 (0.85, 1.01)	0.95 (0.87, 1.03)	**1.15 (1.04, 1.26)**	1.01 (0.93, 1.09)	1.09 (0.99, 1.20)	**1.32 (1.22, 1.43)**	0.97 (0.85, 1.10)	**0.65 (0.50, 0.85)**	**1.13 (1.04, 1.22)**	1.22 (0.82, 1.83)	1.19 (0.71, 1.97)	1.09 (0.99, 1.19)
Non-Sporting/Hounds	**1.31 (1.18, 1.46)**	1.02 (0.91, 1.15)	**0.71 (0.62, 0.81)**	0.91 (0.82, 1.01)	0.91 (0.78, 1.05)	**0.68 (0.61, 0.76)**	**1.97 (1.66, 2.34)**	0.87 (0.59, 1.28)	**1.13 (1.03, 1.24)**	0.72 (0.45, 1.15)	**0.22 (0.12, 0.44)**	0.96 (0.85, 1.07)
Terrier/Hounds	1.06 (0.96, 1.17)	**1.17 (1.06, 1.30)**	1.09 (0.98, 1.21)	1.05 (0.96, 1.14)	**1.19 (1.05, 1.35)**	0.96 (0.88, 1.05)	**1.49 (1.27, 1.73)**	**0.63 (0.45, 0.88)**	**0.69 (0.64, 0.75)**	**0.45 (0.30, 0.68)**	0.72 (0.49, 1.06)	0.93 (0.84, 1.03)
Toys/Hounds	**1.51 (1.35, 1.68)**	1.00 (0.89, 1.12)	**0.82 (0.73, 0.93)**	**0.66 (0.59, 0.73)**	**0.56 (0.48, 0.66)**	**0.47 (0.42, 0.52)**	**3.25 (2.76, 3.82)**	1.10 (0.76, 1.59)	**0.73 (0.66, 0.80)**	1.33 (0.87, 2.03)	**0.29 (0.16, 0.53)**	0.89 (0.80, 1.00)
Utility/Hounds	0.98 (0.88, 1.09)	**1.12 (1.00, 1.24)**	**0.86 (0.77, 0.96)**	**1.16 (1.06, 1.28)**	**1.54 (1.35, 1.75)**	**1.25 (1.14, 1.37)**	**0.60 (0.49, 0.72)**	**0.64 (0.44, 0.93)**	0.99 (0.91, 1.08)	**0.49 (0.31, 0.77)**	**0.50 (0.32, 0.78)**	1.00 (0.90, 1.12)
Working Dogs/Hounds	0.93 (0.84, 1.02)	1.03 (0.93, 1.13)	0.98 (0.88, 1.09)	1.04 (0.95, 1.14)	**1.48 (1.31, 1.68)**	**1.20 (1.09, 1.31)**	**1.17 (1.00, 1.38)**	0.84 (0.60, 1.17)	**0.77 (0.71, 0.83)**	0.78 (0.53, 1.16)	**0.49 (0.32, 0.74)**	0.99 (0.90, 1.10)
Terrier/Non-Sporting	**0.81 (0.75, 0.87)**	**1.15 (1.06, 1.25)**	**1.53 (1.40, 1.69)**	**1.15 (1.07, 1.24)**	**1.32 (1.18, 1.46)**	**1.41 (1.30, 1.53)**	**0.75 (0.68, 0.84)**	**0.73 (0.54, 0.98)**	**0.61 (0.57, 0.66)**	**0.62 (0.42, 0.92)**	**3.21 (1.77, 5.81)**	0.97 (0.90, 1.06)
Toys /Non-Sporting	**1.15 (1.05, 1.25)**	0.98 (0.88, 1.08)	**1.16 (1.03, 1.30)**	**0.72 (0.66, 0.80)**	**0.62 (0.53, 0.72)**	**0.68 (0.61, 0.76)**	**1.65 (1.47, 1.85)**	1.27 (0.91, 1.77)	**0.65 (0.59, 0.70)**	**1.86 (1.25, 2.76)**	1.30 (0.61, 2.74)	0.94 (0.84, 1.04)
Utility/Non-Sporting	**0.75 (0.69, 0.81)**	**1.09 (1.00, 1.19)**	**1.21 (1.09, 1.34)**	**1.28 (1.18, 1.39)**	**1.70 (1.52, 1.90)**	**1.83 (1.68, 1.99)**	**0.30 (0.26, 0.35)**	0.74 (0.53, 1.03)	**0.88 (0.82, 0.94)**	0.68 (0.44, 1.04)	**2.20 (1.17, 4.16)**	1.05 (0.96, 1.15)
Working Dogs/Non-Sporting	**0.71 (0.65, 0.76)**	1.00 (0.92, 1.09)	**1.38 (1.25, 1.52)**	**1.15 (1.07, 1.24)**	**1.63 (1.47, 1.81)**	**1.76 (1.62, 1.90)**	**0.60 (0.53, 0.66)**	0.97 (0.72, 1.30)	**0.68 (0.64, 0.73)**	1.09 (0.76, 1.58)	**2.17 (1.17, 3.99)**	1.04 (0.95, 1.13)
Toys/Terrier	**1.42 (1.32, 1.53)**	**0.85 (0.78, 0.92)**	**0.75 (0.69, 0.83)**	**0.63 (0.58, 0.68)**	**0.47 (0.41, 0.53)**	**0.48 (0.44, 0.53)**	**2.19 (2.00, 2.40)**	**1.75 (1.33, 2.30)**	1.05 (0.98, 1.13)	**2.98 (2.15, 4.14)**	**0.40 (0.24, 0.68)**	0.96 (0.88, 1.04)
Utility/Terrier	**0.92 (0.87, 0.99)**	0.95 (0.89, 1.01)	**0.79 (0.73, 0.85)**	**1.11 (1.05, 1.18)**	**1.29 (1.20, 1.39)**	**1.29 (1.22, 1.37)**	**0.40 (0.35, 0.46)**	1.02 (0.77, 1.34)	**1.43 (1.35, 1.52)**	1.09 (0.76, 1.57)	**0.69 (0.49, 0.96)**	**1.08 (1.01, 1.15)**
Working Dogs/Terrier	**0.87 (0.83, 0.93)**	**0.87 (0.82, 0.93)**	**0.90 (0.84, 0.95)**	1.00 (0.95, 1.05)	**1.24 (1.16, 1.33)**	**1.24 (1.18, 1.31)**	**0.79 (0.73, 0.86)**	**1.34 (1.07, 1.67)**	**1.11 (1.06, 1.17)**	**1.76 (1.32, 2.34)**	**0.68 (0.50, 0.90)**	**1.06 (1.00, 1.13)**
Utility/Toys	**0.65 (0.60, 0.71)**	**1.12 (1.02, 1.22)**	1.04 (0.94, 1.15)	**1.77 (1.62, 1.92)**	**2.75 (2.42, 3.13)**	**2.68 (2.44, 2.94)**	**0.18 (0.16, 0.21)**	**0.58 (0.43, 0.80)**	**1.36 (1.26, 1.47)**	**0.37 (0.25, 0.53)**	1.70 (0.96, 3.00)	**1.12 (1.03, 1.23)**
Working Dogs/Toys	**0.62 (0.57, 0.66)**	1.03 (0.95, 1.12)	**1.19 (1.09, 1.30)**	**1.59 (1.46, 1.72)**	**2.65 (2.34, 3.00)**	**2.57 (2.35, 2.81)**	**0.36 (0.33, 0.40)**	**0.76 (0.59, 1.00)**	1.05 (0.98, 1.13)	**0.59 (0.44, 0.80)**	1.67 (0.97, 2.87)	**1.11 (1.02, 1.21)**
Working Dogs/Utility	0.95 (0.88, 1.01)	**0.92 (0.86, 0.98)**	**1.14 (1.06, 1.23)**	**0.90 (0.85, 0.95)**	0.96 (0.89, 1.04)	0.96 (0.90, 1.02)	**1.97 (1.73, 2.24)**	**1.31 (1.00, 1.71)**	**0.78 (0.73, 0.82)**	**1.61 (1.14, 2.27)**	0.98 (0.68, 1.43)	0.99 (0.92, 1.06)

The first number is the odds ratio, and the two numbers in the brackets are the CI. Bold: the odds ratio (CI) was marked as statistically significant (*p* < 0.05) when the CI did not cover 1.00. ^a^ A person was reported to have abused an animal.

## Appendix A

**Table A1:** Description of each complaint code alleging a welfare issue.

Complaint Code	Description
Abandonment	An animal was abandoned/left by the owner either at their abode or somewhere else such as in the bush.
Baiting/poisoning	An animal was poisoned or planned to be poisoned.
Cause captive animal to be injured/killed by dog	A person let a captive animal be injured/killed by a dog.
Cruelty	A person was reported to have abused an animal.
Dog fighting or other prohibited offence	A person was reported as allowing dogs to fight or conducting other specifically prohibited acts.
Emergency relief	Emergency relief is required for an animal left unattended because its owner experienced an emergency (e.g., flood or being hit by a car).
Hot animal in car	An animal was left unattended in a car during hot weather.
Insufficient food and/or water	An animal has insufficient food and/or water.
Keep or use animal for blooding/coursing a dog	A person used a live bait for blooding/coursing a dog.
Knowingly allow an animal to kill/injure another	A person allows one animal to kill/injuring another one, and does nothing to stop them.
No exercise/confined/tethered	An animal is confined or tethered and not given a suitable amount of exercise.
No shelter	An animal is not provided with suitable shelter provisions.
No treatment	An animal did not receive appropriate medical treatment when needed.
Overcrowding	The number of animals was too high for the living space provided.
Poor dog condition	The general condition of an animal is poor. (e.g., messy/matted coat, pussy eyes, etc.)
Poor living condition	The living environment of the animal is poor.
Prohibition order breached	An owner violated a prohibition order. ^a^
Tail docking or other surgical procedure	Tail docking or other surgical procedure (e.g., declaw removal, etc.) was conducted on an animal.

^a^ Prohibition order: A prohibition order is given by the court when a person convicted of an animal welfare offense must not possess any or specific animal for a prescribed period of time [2].

**Table A2:** Breed list.

Comment from the Public	Breed List	Breed Group
Affenpinscher	Affenpinscher	Toys
Afghan hound	Afghan hound	Hounds
Airedale terrier	Airedale terrier	Terrier
Akita	Akita	Utility
Alaskan husky	Siberian husky	Utility
Alaskan malamute	Alaskan malamute	Utility
American bulldog	American bulldog	Non-sporting
American foxhound	Foxhound	Hounds
American pit bull terrier	Pit bull terrier	Terrier
American Staffordshire terrier	Staffordshire terrier	Terrier
American water spaniel	American water spaniel	Gundogs
Anatolian shepherd dog	Anatolian shepherd dog	Utility
Australian bandog	Cross breed	N-RB
Australian bulldog Australian bulldog cross	Australian bulldog	Non-sporting
Australian cattle dog	Australian cattle dog	Working dogs
Australian koolie	Coolie/koolie	N-RB
Australian sheepdog	Australian sheepdog	Working dogs
Australian shepherd	Australian shepherd	Working dogs
Australian silky terrier	Australian silky terrier	Toys
Australian stumpy tail cattle dog	Australian stumpy tail cattle dog	Working dogs
Australian terrier	Australian terrier	Terrier
Bandogge mastiff	Cross breed	N-RB
Basenji	Basenji	Hounds
Basset fauve de Bretagne	Basset fauve de Bretagne	Hounds
Basset hound	Basset hound	Hounds
Beagle	Beagle	Hounds
Bearded collie	Bearded collie	Working dogs
Bedlington terrier	Bedlington terrier	Terrier
Belgian shepherd	Belgian shepherd	Working dogs
Belgian shepherd—Groenendael		Working dogs
Belgian shepherd—Laekenois		Working dogs
Belgian shepherd—Malinois		Working dogs
Belgian shepherd—Tervueren		Working dogs
Bernese mountain dog	Bernese mountain dog	Utility
Bichon frise	Bichon frise	Toys
Bloodhound	Bloodhound	Hounds
Bluetick coohound	Bluetick coohound	Hounds
Border collie Border collie × Labrador Border collie, miniature	Border collie	Working dogs
Border terrier	Border terrier	Terrier
Borzoi	Borzoi	Hounds
Boston terrier	Boston terrier	Non-sporting
Bouvier des flandres	Bouvier des Flandres	Working dogs
Boxer Boxer cross Boxer × bullmastif Boxer × American Staffordshire terrier	Boxer	Utility
Bracco Italiano	Bracco Italiano	Gundogs
Briard	Briard	Working dogs
British bulldog	British bulldog	Non-sporting
Brittany	Brittany	Gundogs
Bull Arab Bull Arab × greyhound	Bull Arab	N-RB
Bull terrier Bull terrier cross Bull Terrier, miniature	Bull terrier	Terrier
Bulldog Bulldog cross	British bulldog	Non-sporting
Bullmastiff Bullmastiff cross Bullmastiff × wolfhound × Great Dane	Bullmastiff	Utility
Cane corso (Italian mastiff)	Cane corso	Utility
Canaan dog	Canaan dog	Non-sporting
Cairn terrier	Cairn terrier	Terrier
Cattle dog Cattle dog cross	Australian cattle dog	Working dogs
Cavalier King Charles spaniel	Cavalier King Charles spaniel	Toys
Central Asian shepherd dog	Central Asian shepherd dog	Utility
Cesky terrier	Cesky terrier	Terrier
Chesapeake Bay retriever	Chesapeake Bay retriever	Gundogs
Chihuahua Chihuahua cross Chihuahua × Jack Russell Long hair chihuahua	Chihuahua	Toys
Chinese crested dog Chinese crested dog—powder puff	Chinese crested dog	Toys
Chow chow	Chow chow	Non-sporting
Clumber spaniel	Clumber spaniel	Gundogs
Cocker spaniel Cocker spaniel, American Cocker spaniel, English	Cocker spaniel	Gundogs
Collie Collie rough Collie smooth	Collie	Working dogs
Corgi Corgi, Cardigan Welsh Corgi, Pembroke Welsh Corgi × fox terrier	Corgi	Working dogs
Coton de Tulear	Coton de Tulear	Toys
Cross breed	Cross breed	N-RB
Curly coated retriever	Curly coated retriever	Gundogs
Dachshund Dachshund, long-haired Dachshund, miniature	Dachshund	Hounds
Dalmatian Dalmatian cross	Dalmatian	Non-sporting
Dandie dinmont terrier	Dandie dinmont terrier	Terrier
Deerhound	Deerhound	Hounds
Dingo Dingo cross	Cross breed	N-RB
Dobermann	Dobermann	Utility
Dogue de Bordeaux	Dogue de Bordeaux	Utility
Dunker	Dunker	Hounds
Dutch shepherd	Dutch shepherd	Working dogs
English foxhound	Foxhound	Hounds
English pointer	English pointer	Gundogs
English mastiff	English mastiff	Utility
English setter	English setter	Gundogs
English springer spaniel	Springer spaniel	Gundogs
English toy terrier	English toy terrier	Toys
Field spaniel	Field spaniel	Gundogs
Finnish lapphund	Finnish lapphund	Working dogs
Flat coated retriever	Flat coated retriever	Gundogs
Formosan mountain dog (Taiwan dog)	Formosan mountain dog	Utility
Fox terrier Fox terrier, smooth	Fox terrier	Terrier
Foxhound	Foxhound	Hounds
French bulldog	French bulldog	Non-sporting
German coolie	Coolie/koolie	N-RB
German hunting terrier	German hunting terrier	Terrier
German pinscher	German pinscher	Utility
German shepherd German shepherd cross	German shepherd	Working dogs
German shorthaired pointer	German shorthaired/wirehaired pointer	Gundogs
German spitz	Spitz	Non-sporting
German wirehaired pointer	German shorthaired/wirehaired pointer	Gundogs
Glen of Imaal terrier	Glen of Imaal terrier	Terrier
Golden retriever	Golden retriever	Gundogs
Gordon setter	Gordon setter	Gundogs
Great Dane Great dane × bull Arab Great dane × bullmastiff	Great Dane	Non-sporting
Great Pyrenees	Great Pyrenees	Working dogs
Greater Swiss mountain dog	Greater Swiss mountain dog	Working dogs
Greyhound	Greyhound	Hounds
Griffon Bruxellois	Griffon Bruxellois	Toys
Harrier	Harrier	Hounds
Havanese	Havanese	Toys
Hungarian vizsla	Hungarian vizsla	Gundogs
Husky Husky cross	Siberian husky	Utility
Ibizan hound	Ibizan hound	Hounds
Irish red and white setter	Irish setter	Gundogs
Irish setter		Gundogs
Irish terrier	Irish terrier	Terrier
Irish water spaniel	Irish water spaniel	Gundogs
Irish wolfhound	Irish wolfhound	Hounds
Italian greyhound	Italian greyhound	Toys
Italian spinone	Italian spinone	Gundogs
Jack Russell terrier	Jack Russell terrier	Terrier
Japanese chin	Japanese chin	Toys
Japanese spitz	Spitz	Non-sporting
Kangal shepherd dog	Kangal shepherd dog	Utility
Keeshond	Keeshond	Non-sporting
Kelpie Kelpie cross Kelpie × staffordshire terrier Kelpie × border collie Kelpie × cattle dog Kelpie × labrador Kelpie × dingo	Kelpie	Working dogs
Kerry blue terrier	Kerry blue terrier	Terrier
King Charles spaniel	King Charles spaniel	Toys
Kuvasz	Kuvasz	Working dogs
Labrador retriever Labrador retriever cross Labradoodle	Labrador retriever	Gundogs
Lagotto Romagnolo	Lagotto Romagnolo	Gundogs
Lakeland terrier	Lakeland terrier	Terrier
Large Munsterlander	Large Munsterlander	Gundogs
Leonberger	Leonberger	Utility
Large terrier cross	Terrier	Terrier
Lancashire heeler	Lancashire heeler	Working dogs
Lhasa apso	Lhasa apso	Non-sporting
Louisiana Catahoula leopard dog	Louisiana Catahoula leopard dog	Working dogs
Löwchen	Löwchen	Toys
Lurcher	Cross breed	N-RB
Maltese Maltese cross	Maltese	Toys
Manchester terrier	Manchester terrier	Terrier
Maremma sheepdog	Maremma sheepdog	Working dogs
Mastiff Mastiff cross Mastiff × bull Arab	Mastiff	Utility
Medium terrier Medium terrier cross	Terrier	Terrier
Miniature fox terrier	Fox Terrier	Terrier
Miniature pinscher	Miniature pinscher	Toys
Neapolitan mastiff	Neapolitan mastiff	Utility
New Zealand huntaway	New Zealand huntaway	Working dogs
Newfoundland	Newfoundland	Utility
Norfolk terrier	Norfolk terrier	Terrier
North Queensland bullhound	Cross breed	N-RB
Norwegian elkhound	Norwegian elkhound	Hounds
Norwich terrier	Norwich terrier	Terrier
Nova Scotia duck tolling retriever	Nova Scotia duck tolling retriever	Gundogs
Old English sheepdog	Old English sheepdog	Working dogs
Papillon	Papillon	Toys
Parson Russell terrier	Parson Russell terrier	Terrier
Pekingese	Pekingese	Toys
Peruvian hairless dog	Peruvian hairless dog	Hounds
Petit basset griffon vendeen	Petit basset griffon vendeen	Hounds
Pharaoh hound	Pharaoh hound	Hounds
Pit bull terrier	Pit bull terrier	Terrier
Pig dog	Cross breed	Terrier
Pointer	Pointer	Gundogs
Polish lowland sheepdog	Polish lowland sheepdog	Working dogs
Pomeranian	Pomeranian	Toys
Poodle Poodle toy Poodle miniature Poodle standard Poodle × shih tzu	Poodle	Non-sporting
Portugese podengo	Portugese podengo	Hounds
Portuguese water dog	Portuguese water dog	Utility
Pug	Pug	Toys
Puli	Puli	Working dogs
Prague ratter	Cross breed	N-RB
Pyrenean mastiff	Pyrenean mastiff	Utility
Pyrenean mountain dog	Pyrenean mountain dog	Utility
Rhodesian ridgeback	Rhodesian ridgeback	Hounds
Rottweiler Rottweiler × mastiff	Rottweiler	Utility
Russian black terrier	Russian black terrier	Utility
Saint bernard	Saint bernard	Utility
Saluki	Saluki	Hounds
Samoyed	Samoyed	Utility
Sarplaninac	Sarplaninac	Utility
Schipperke	Schipperke	Non-sporting
Schnauzer Schnauzer, miniature Schnauzer, standard Schnauzer, giant	Schnauzer	Utility
Scottish terrier	Scottish terrier	Terrier
Sealyham terrier	Sealyham terrier	Terrier
Shar pei Shar Pei cross	Shar pei	Non-sporting
Shetland sheepdog	Shetland sheepdog	Working dogs
Shiba inu	Shiba inu	Utility
Shih tzu Shih tzu × maltese	Shih tzu	Non-sporting
Siberian husky	Siberian husky	Utility
Skye terrier	Skye terrier	Terrier
Sloughi	Sloughi	Hounds
Small terrier cross	Terrier	Terrier
Smithfield cattle dog	Cross breed	N-RB
Soft coated wheaten terrier	Soft coated wheaten terrier	Terrier
Spaniel	Spaniel	Gundogs
Spanish water dog	Spanish water dog	Gundogs
Spitz	Spitz	Non-sporting
Spoodle	Cocker spaniel	Gundogs
Staffordshire bull terrier Staffordshire bull terrier × labrador	American Staffordshire bull terrier	Terrier
Staghound	Staghound	N-RB
Swedish vallhund	Swedish vallhund	Working dogs
Tenterfield terrier	Tenterfield terrier	Terrier
Terrier	Terrier	Terrier
Thai ridgeback	Thai ridgeback	Hounds
Tibetan mastiff	Tibetan mastiff	Utility
Tibetan spaniel	Tibetan spaniel	Toys
Tibetan terrier	Tibetan terrier	Non-sporting
Timber shepherd	Cross breed	N-RB
Weimaraner	Weimaraner	Gundogs
Welsh springer spaniel	Springer spaniel	Gundogs
Welsh terrier	Welsh terrier	Terrier
West highland white terrier	West highland white terrier	Terrier
Whippet	Whippet	Hounds
White Swiss shepherd dog	White Swiss shepherd dog	Working dogs
Wirehaired fox terrier	Fox terrier	Terrier
Xoloitzcuintle	Xoloitzcuintle	Non-sporting
Yorkshire terrier	Yorkshire terrier	Toys

ANKC: Australian National Kennel Council (http://ankc.org.au/); AMKC: American Kennel Club (https://www.akc.org/dog-breeds/); UKC: United Kennel Club (https://www.ukcdogs.com/breed-standards); NZKC: New Zealand Kennel Club (https://www.dogsnz.org.nz/home/home).
